# Etiology, Diagnostic, and Rehabilitative Methods for Children with Central Auditory Processing Disorders—A Scoping Review

**DOI:** 10.3390/audiolres14040062

**Published:** 2024-08-21

**Authors:** Andrzej Karol Konopka, Anna Kasprzyk, Julia Pyttel, Lechosław Paweł Chmielik, Artur Niedzielski

**Affiliations:** 1 Oticon Polska Sp. z o.o., Aleja Jana Pawła II 22, 00-133 Warsaw, Poland; 2 Department of Pediatric Otolaryngology, Centre of Postgraduate Medical Education, 01-813 Warsaw, Polandl.p.chmielik@chmielik.pl (L.P.C.); arturniedzielski@wp.pl (A.N.); 3 Department of Pediatric ENT, The Children’s Hospital in Dziekanow Lesny, 05-092 Dziekanow Lesny, Poland; 4 ACS Audika Sp. z o.o., 00-133 Warsaw, Poland; juyp@audika.pl

**Keywords:** central auditory processing disorder, auditory processing disorders, CAPD, APD, children

## Abstract

APD (auditory processing disorders) is defined as difficulties in processing auditory stimuli within the central nervous system, with normative physical hearing and intellectual disabilities excluded. The scale of this phenomenon among children and adolescents and the need to raise awareness of its occurrence prompted the authors to review currently available diagnostic and therapeutic methods, as well as outline future directions for addressing children affected by APD.

## 1. Introduction

Central auditory processing disorder (APD) refers to impairments in processing auditory stimuli due to central nervous system abnormalities despite intact peripheral auditory structures. The disorder manifests through a diverse array of symptoms. These processes underlie fundamental auditory skills such as sound localization, sound discrimination, pattern recognition, temporal analysis of sound signals, and temporal integration of sounds [1].

In individuals with APD, their physical hearing is intact, but the “impairment” occurs in the central nervous part of the auditory system, leading to improper processing of auditory stimuli. This can significantly impact the daily functioning of the individual. The brain of a person with APD is unable to recognize and interpret sounds, including speech sounds, despite having normal physical hearing [2].

In terms of prevalence, around 3–5% of school-aged children are affected by an APD [3]. According to various studies, DiMaggio and Geffner reported a rate of 12% [4], Katz indicated a rate of 20%, and Skarżyński [5] confirmed a rate of 11%.

The topic of diagnostic methods and rehabilitation for APD and their standardization is the focus of attention for many national and international research groups around the world, including the American Academy of Audiology [6], the American Speech–Language–Hearing Association [2], the British Society of Audiology [7], the California Speech–Language–Hearing Association [8], the Canadian Interorganizational Steering Group for Speech–Language Pathology and Audiology [9], and the German Society for Phoniatrics and Pediatric Audiology [10,11]. The exchange of experiences and results from ongoing research could lead to the establishment of a unified approach to the diagnosis and rehabilitation of APD on a European [12] and even global scale.

Within the ICD-10-CM, APD is categorized under “other disorders of auditory perception” (code H93.25), characterized by challenges in identifying, discriminating, distinguishing, and processing auditory information within the central nervous system [12].

APD has been classified under group AB5Y in the latest ICD-11 classification that came into effect widely in 2022.

The aim of this study was to review the literature on auditory processing disorder with a focus on pediatric patients. This article provides a detailed overview of the etiology of APD in children, its correlated disorders, and diagnostic and therapeutic strategies. For this review, the authors searched primary, secondary, and tertiary sources (published between 1986 and 2024) using the PubMed, Google Scholar, and Scopus databases. The following keywords were used: auditory processing disorder or central auditory processing disorder or APD or CAPD and children. The final research for this article was conducted in July 2024.

## 2. Classification of APD

APD can be classified into several categories based on different criteria, such as etiology, symptoms, or predominant difficulties presented by individuals.

Three categories of APD are categorized by their etiology, as outlined by Keith [13]:

Developmental APD is present in children exhibiting APD symptoms, with typical hearing sensitivity and no identified alternative cause for hearing difficulties [14]. These implications could persist into adulthood.

Acquired APD is often linked to prenatal or postnatal occurrences such as strokes, oxygen deprivation, premature birth, elevated bilirubin levels, cytomegaly, toxoplasmosis, or infections [15].

Secondary APD arises following conductive hearing loss because of prolonged middle ear infections during development [16,17].

Different clinical profiles are identified based on the dominant symptoms. Phonological processing disorders (decoding deficit) involve challenges at the phonetic and phonological levels, impacting reading, writing, and speech functions. Auditory attention deficit and difficulties in noise perception include struggles with understanding speech in unfavorable acoustic settings, processing distorted or rapid speech, concentration issues, and memory challenges. Auditory–visual integration disorders (integration deficit) center around difficulties in merging auditory and visual information and concern nonverbal speech characteristics [18].

## 3. Etiology of APD

The etiology of APD is multifaceted and lacks a clear definition. APD can stem from various causes like premature birth [19], perinatal oxygen deprivation, neuroinfections and viral infections during infancy [20], CNS injuries and tumors, strokes, toxic exposure, prolonged or repeated periods of auditory deprivation (such as recurrent otitis media), notably during crucial stages of auditory development [21], and genetic predispositions [22,23]. Currently, there is limited consensus regarding the exact genesis of APD. The cause of this condition remains unknown in most children diagnosed with APD [24].

According to Senderski [25], the increase in the number of children experiencing auditory perception issues, despite normal hearing sensitivity, can be influenced by excessive stimulation from visual and auditory stimuli (internet, computer games, television, etc.). This excessive stimulation may overwhelm a child’s perceptual abilities, disrupting their acquisition of communication skills. An excess of stimuli impairs the ability to filter and select information, ultimately leading to attention concentration disorders [26].

Recent MRI studies of children with APD indicate changes in structural networks at the regional level of the cerebrum, suggesting the presence of multimodal deficits and the influence of structure–function alterations on their listening difficulties [27].

## 4. Consequences of APD

APD can result in speech delays and reduced comprehension, weaken auditory memory, contribute to articulation issues, induce sensitivity to sounds, and frequently be associated with emotional challenges like low self-esteem, shyness, and behavioral issues [28].

Certain irregularities may become evident in a child during early childhood, but they become increasingly noticeable as the child reaches school age and encounters challenges in learning tasks like reading, writing, distinguishing similar sounds and words, maintaining legible handwriting, spelling accurately, concentrating, following the teacher’s instructions, comprehending longer texts or stories, articulating ideas fluently, and acquiring the skills needed for learning a new language [29]. Children with APD are frequently perceived as less capable, inattentive, distracted, and lazy.

Meanwhile, their issue lies in getting easily fatigued during tasks requiring concentration on auditory stimuli, facing challenges in maintaining attention, and inability to block access to irrelevant and unwanted stimuli, resulting in being easily distracted. This leads to challenges in the emotional and social functioning of children with APD [30].

Children with APD sometimes develop behavioral disorders as they seek their peer group, with their difficulties leading them towards peers with poorer functioning. The combination of APD and behavioral issues presents a significant challenge when working with such patients. Often, children with APD struggle to function well in peer groups as they have difficulty understanding speech when multiple people are talking simultaneously. This can lead them to have only one friend. Additionally, the sound sensitivity frequently seen in children with APD can cause them to avoid school discos and trips, resulting in a lack of shared experiences with peers and adversely affecting their social functioning in this aspect [31].

## 5. Diagnosis of APD

APD is diagnosed when at least one of the higher auditory functions is impaired, including sound localization, sound discrimination, pattern recognition, analysis of temporal sound features (duration, temporal resolution, perception of sequence), understanding distorted speech, or speech in noise [32]. These stimuli hinder the educational process of individuals with APD.

It is crucial for children with APD to be diagnosed promptly for appropriate therapy. To diagnose APD, all types of hearing loss [28] and intellectual disabilities in children must be ruled out [33].

An accurate diagnosis of APD should involve an interdisciplinary team [18] comprising a psychologist (to rule out intellectual disabilities), an audiologist (to conduct physical hearing tests), an educational therapist/speech therapist/sensory integration therapist (to perform tests evaluating higher auditory functions), phoniatrist, and an otolaryngologist/audiology doctor (for the ultimate diagnosis) [33,34].

The diagnostic test batteries evaluating higher auditory functions aim to assess the functionality of the central auditory nervous system (CANS), determine the presence of APD, and describe its parameters to facilitate deficit-specific intervention programming.

Dichotic speech tests are designed to evaluate an individual’s capacity for processing different auditory information presented simultaneously to each ear. In these tests, individuals are tasked with identifying or repeating information presented to each ear separately. The primary aim of these assessments is to gauge hemispheric lateralization for speech and to assess how effectively the brain processes simultaneous auditory input [4,35,36]. Examples include dichotic digit tests, competing sentences tests, and dichotic consonant–vowel tests.

Monaural low-redundancy tests serve to assess an individual’s ability to comprehend speech under challenging conditions by presenting distorted speech signals to one ear. By exposing individuals to degraded speech material, these tests aim to measure their proficiency in understanding and processing speech in complex auditory environments, replicating real-world listening scenarios, and highlighting potential difficulties in speech comprehension [37,38]. Examples include time-compressed speech tests, filtered speech tests, and competing speech tests.

Auditory temporal processing and patterning tests focus on evaluating the temporal analysis of auditory signals, covering aspects such as temporal resolution, sound order perception, temporal integration, and temporal masking. By delving into how well individuals process and differentiate temporal features of sound, these assessments offer valuable insights into an individual’s temporal auditory processing abilities and help identify any potential areas of difficulty [31,37]. Examples include frequency pattern tests, duration pattern tests, and gap detection tests [39].

Binaural interaction tests delve into the interaction between the auditory systems of both ears, assessing the maturity of the auditory system, hemispheric lateralization for speech, and auditory attention. By presenting different verbal stimuli to each ear simultaneously, these tests provide means to evaluate how effectively the brain integrates information from both ears, shedding light on the individual’s overall auditory processing capabilities and any potential challenges present [4,40]. Examples include dichotic listening tests and binaural fusion tests [41].

Auditory discrimination tests are utilized to evaluate an individual’s aptitude for distinguishing between various auditory stimuli. Tasks within these tests may include discriminating between similar sounds, detecting subtle nuances in speech [42], or identifying specific patterns within auditory information. These assessments play a crucial role in pinpointing any difficulties an individual may have in auditory discrimination, which can impact different facets of auditory processing and guide targeted interventions [4,37]. Examples include frequency discrimination tests, intensity discrimination tests, and duration discrimination tests.

It is also possible to perform brief screening tests for APD [43]. A valuable tool for assessing auditory skills is the Scale of Auditory Behavior (SAB) (Table 1). The questionnaire consists of 12 questions related to everyday activities rated on a scale from 1 to 5 (depending on the frequency of occurrence). The sum of all values results in final scores ranging between 12 and 60 points. If the score is less than 35 points, the patient should be referred for APD testing [44].

Validated questionnaires designed for children with APD include the Children’s Auditory Processing Performance Scale (CHAPPS) [45], Fisher’s Auditory Problems Checklist (FAPC) [46], and the Auditory Processing Domains Questionnaire (APDQ) [47]. These tools offer insights into different facets of auditory function, addressing both directly related aspects of hearing (like performance in quiet, optimal conditions, or in noisy environments for CHAPPS) and indirectly related factors (such as attention and memory) [48].

When making a diagnosis, it is necessary to take a holistic approach to the patient. This approach allows for conducting a comprehensive interview with the patient’s legal guardian and a detailed analysis of medical and specialist documentation. Before starting the diagnostic process, it is important to indicate the need to bring and present such documentation to the diagnosing specialist [49].

**Figure 1:**
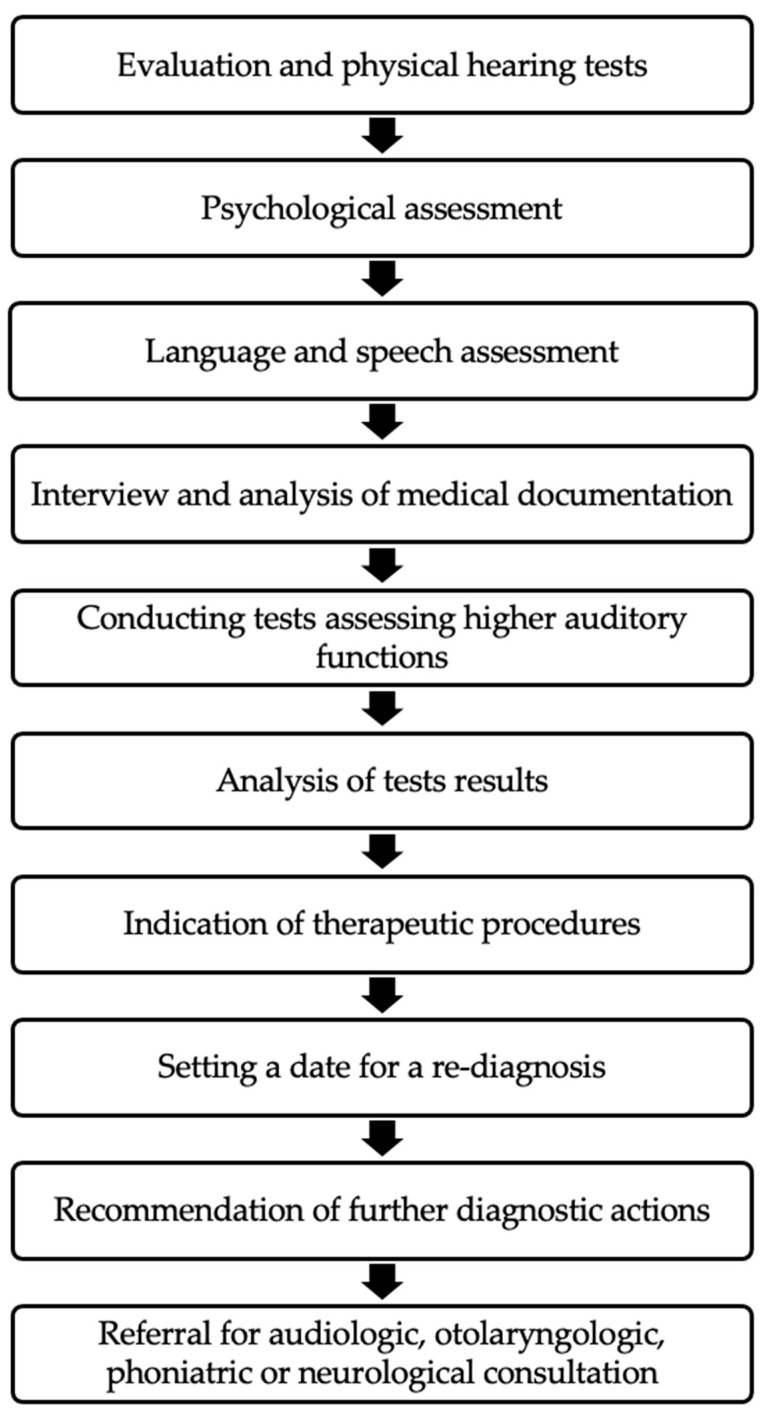
Summary of diagnostic approach of patients with APD [50].

The diagnosis of a patient for APD should consist of the elements shown in Figure 1.

The diagnosis of APD should be conducted in individuals aged 6–7 years, as auditory perception physiologically continues to develop until this age [12].

For some patients with APD, it is necessary to expand the diagnostic process to include phonemic hearing assessment [51]. If a child does not differentiate between sound frequencies, they may struggle with distinguishing phonemes, especially contrasting phonemes that are acoustically similar [52]. An objective tool for phonemic function diagnostic capabilities should be used for this procedure.

After analyzing the research results and based on the child’s free observation in the office, it may be necessary to perform a differential diagnosis of other pathologies that may lead to poor performances on central auditory processing tasks. Recognition of potential lesions in the central nervous system may be helpful for identifying what central auditory processing is afflicted. Due to the complex nature of brain function, APD may accompany other developmental disorders such as attention deficit disorder (ADD) or attention deficit hyperactivity disorder (ADHD), specific learning difficulties, and dyslexia. To differentiate between two or more conditions presenting with similar symptoms, a multidisciplinary approach is needed [24,53,54].

The lack of a standardized test battery used in the diagnostic process leads to much confusion. Selection of the tests used to diagnose APD should be tailored according to the complaints and information obtained from patients and their parents [24].

## 6. Rehabilitation of Children with APD

Therapy for children with auditory processing disorder (APD) typically involves a multidisciplinary approach tailored to the individual needs of the child [18]. The common components of therapy for APD include:

1. Auditory Training: exercises to improve the brain’s ability to process and interpret auditory information [55].
2. Cognitive Behavioral Therapy: addressing any emotional or behavioral challenges associated with APD.
3. Speech and Language Therapy: targeted intervention to improve speech, language, and communication skills [38].
4. Environmental Modifications (including classroom-based strategies): creating a conducive environment to reduce auditory distractions and enhance listening skills [56].
5. Assistive Listening Devices: using wireless technology like FM systems [57,58].

It is important for therapy to be individualized based on the specific challenges and strengths of each child with APD. Working closely with audiologists, speech–language pathologists, psychologists, and educators can help create an effective treatment plan for children with APD [18].

As part of training to correct auditory deficits, various methods and auditory training are proposed, including:

- Tomatis therapy, developed by Dr. Alfred Tomatis, utilizes sound-based techniques to retrain the brain’s auditory processing abilities. This therapy revolves around the use of carefully filtered music and sounds to target specific frequencies, aiming to stimulate and enhance the auditory system. Through a method called “audio-vocal feedback,” individuals participate in listening exercises designed to improve various cognitive functions such as attention, language skills, and emotional regulation. Customized sessions involve wearing headphones equipped with bone conduction transducers to deliver the specialized music directly to the inner ear. Tomatis therapy is commonly employed to address conditions like auditory processing disorders, learning difficulties, and speech delays. The treatment progresses systematically based on individual assessments, gradually working towards optimizing cognitive and sensory processing functions [59].
- Individual sound stimulation by Johansen’s method—this method was created by Kjeld Johansen, a Danish teacher and psychologist. Johansen’s Individual Sound Stimulation Method is based on specially synthesized music recorded on a CD. The therapeutic program in the form of individually filtered instrumental music is recorded on CDs. The patient receives a practice CD at home and listens to it daily for about 10 min with headphones [60].
- Warnke method—this therapy is conducted using special devices. Most of the tasks performed by the therapy participant resemble simple computer games. Portable devices are used, which can be used at home or in a therapy center [61,62,63].
- SPPS-S method (stimulation of polymodal sensory perception by Henryk Skarżyński’s method)—a core assumption of this method is the ability to use auditory training in everyday life. The miniature sensory perception stimulator is a portable device constructed using state-of-the-art microelectronic technology. The stimulator allows for the implementation of various therapy programs based on the use of digital sound processing algorithms. Classical algorithms are based on the concept of the “electronic ear,” while others transform sounds in a way that listening to them affects the improvement of auditory lateralization. The “electronic ear” mainly influences the improvement of the micro-muscle motorics of the middle ear, while algorithms related to improving lateralization are intended to mitigate the effects of abnormalities in this area [64].
- Forbrain headphones—the main assumption used in Forbrain headphones is the application of an audio–vocal loop. Therapy is conducted at the patient’s home or at the therapist’s office [65].
- Interactive Metronome—developed in 1992 by James Cassily. Therapy using this method is performed with special computer software using additional therapeutic aids: a button trigger, a touch mat, and moving switches. The patient’s task is to perform specific tasks to the rhythm of the music. The therapy has several forms of implementation: in therapy centers or with a specially developed home module [66].
- iLS—Integrated Listening System—this auditory training is developed based on Alfred Tomatis’ method. A portable auditory therapy device and a balance and coordination program were developed for the method’s implementation. The therapy can be conducted in a therapy center or at the patient’s home [67].
- Fast ForWord Therapy—this therapy is based on Ernst Poppel’s concept of time perception. Fast ForWord therapy is performed using a developed computer program. It consists of several programs: a basic program, a language program, and a reading and writing program. The therapy is carried out at the patient’s home [68].
- Therapeutic listening—this auditory stimulation is based on sensory integration principles. The therapy involves daily listening to prepared sound material tailored to the specific patient. This therapy is carried out at the patient’s home [69].
- FM-based neurorehabilitation method—is based on the FM system [70], which is ordered unilaterally for the patient’s right ear. This method brings about immediate effects by improving SNR and focusing attention on auditory stimuli through the implementation of an assisting device for hearing [71].
- The type and method of therapy should be individually tailored to the needs and abilities of each patient [72]. It is crucial for a child with APD to receive appropriate educational and functional recommendations that enable them to function better in daily life. These recommendations should include both adjustments to be made in the educational setting (such as creating suitable listening conditions by reducing unnecessary noise, seating the student in the front row—ideally directly in front of the teacher, etc.) and guidance on how to communicate with the child to ensure understanding (such as maintaining eye contact while speaking, using additional repetitions and explanations, making sure the verbal message is correctly understood by the child, etc.) [73].
- Children with APD often struggle with reduced self-esteem, highlighting the importance of providing emotional support and recognizing their efforts. In therapy for APD, teachers and parents play significant roles in understanding the auditory challenges children face. Improvement in the patient’s ability to communicate effectively is the main goal of therapeutic management. Guidelines suggest strategies such as preceding auditory information with visual cues to enhance attention, maintaining eye contact, slow speaking rate, and preparing children for upcoming important information [18]. Additional recommendations include providing notes in advance, summarizing key points, creating a quiet learning environment, and using an FM system to reduce noise distractions [57]. Effective communication methods, repetition of key information, and using simple language structures are encouraged. Recognizing signs of fatigue and allowing for breaks when concentration wanes are crucial for optimizing learning outcomes for children with APD.

## 7. Discussion and Future Directions

APD poses a notable challenge for children, impacting their school performance and daily life. Many teachers are unaware of these auditory issues, especially the more complex aspects. Another challenge is forming teams capable of diagnosing and supporting children with APD through therapy. Looking ahead, an important challenge for the future remains the issue of objectifying diagnostic tests and the results obtained from them depending on the country, language, and cultural and educational backgrounds. A universal tool or platform that enables the creation of a unified diagnostic–rehabilitation path for patients with APD would address this challenge [12]. In the past, APD was often misdiagnosed as ADHD, anxiety disorders, and dyslexia. As symptoms of these disorders overlap, APD is easy to confuse with other conditions, which further complicates the diagnosis and treatment. The solution to improving outcomes in such cases is comprehensive care provided by a multidisciplinary team. Nowadays, misdiagnoses of APD occur less often due to the greater awareness of the importance of differential diagnosis [10]. Given the importance of the above-mentioned issues, exploring new diagnostic methods and effective therapies, including behavioral tests and scientific research, is a natural progression.

## Figures and Tables

**Table 1:** Scale of Auditory Behavior (SAB) questionnaire.

Behavior Items	Very Often	Often	Sometimes	Rarely	Never
Has difficulty hearing or understanding speech in noise.	1	2	3	4	5
2. Has difficulty understanding fast or quiet speech.	1	2	3	4	5
3. Has difficulty following verbal instructions.	1	2	3	4	5
4. Has difficulty distinguishing and identifying speech sounds.	1	2	3	4	5
5. Responds inappropriately to information provided auditorily.	1	2	3	4	5
6. Has hearing impairment.	1	2	3	4	5
7. Asks for information to be repeated.	1	2	3	4	5
8. Easily gets distracted.	1	2	3	4	5
9. Has difficulty learning, achieves poorer academic results.	1	2	3	4	5
10. Has a short attention span.	1	2	3	4	5
11. Gets distracted, daydreams, e.g., in class.	1	2	3	4	5
12. Is disorganized, cannot plan their actions.	1	2	3	4	5
Score: __________ (sum of items circled)					

## Data Availability

No new data were created or analyzed in this study. Data sharing is not applicable to this article.
